# Human T-cell lymphotropic virus type 1 infection among U.S. Thalassemia patients

**DOI:** 10.1186/1742-4690-11-S1-P54

**Published:** 2014-01-07

**Authors:** William M Switzer, Anupama Shankar, Sean R Trimble, Alexis A Thompson, Patricia J Giardina, Alan R Cohen, Thomas D Coates, Elliott Vichinsky, Ellis J Neufeld, Jeanne M Boudreaux, Walid Heneine

**Affiliations:** 1 Laboratory Branch, Division of HIV/AIDS Prevention, National Center for HIV, Hepatitis, STD, and TB Prevention, Centers for Disease Control and Prevention, Atlanta, GA, USA; 2 Epidemiology and Surveillance Branch, Division of Blood Disorders, National Center on Birth Defects and Developmental Disabilities, Centers for Disease Control and Prevention, Atlanta, GA, USA; 3 Ann & Robert H. Lurie Children’s Hospital of Chicago, Chicago, IL, USA; 4 Weill Medical College of Cornell University, New York NY, USA; 5 Children’s Hospital of Philadelphia, Philadelphia, PA, USA; 6 Children’s Hospital of Los Angeles, Los Angeles, CA, USA; 7 Children’s Hospital of Oakland, Oakland, CA, USA; 8 Children’s Hospital Boston, Boston, MA, USA; 9 Children’s Healthcare of Atlanta, Atlanta, GA, USA

## 

Thalassemia is an inherited genetic disorder requiring multiple transfusions to treat anemia caused by low hemoglobin levels. Thus, thalassemia patients are at risk for infection with blood-borne pathogens, including human T-cell lymphotropic viruses (HTLV) that are transmitted by transfusion of cellular blood products. Here, we examined the prevalence of HTLV among 234 US thalassemia patients using sera collected in 2008. Sera were tested for antibodies to HTLV-1/2 using EIA and a confirmatory Western blot (WB) that differentiates between HTLV-1 and HTLV-2. Demographic and clinical information were collected at study enrollment, including HIV and HCV status. Three patients (1.3%) were WB-positive, two were HTLV-1 and one could not be serotyped as HTLV-1/2. All three HTLV-positive persons were HIV-1 negative and only one was HCV seropositive. The HTLV seroprevalence was higher than that of HIV-1 (0.85%) and lower than HCV (18.8%) in this population. All three patients (ages 26-46 years) were diagnosed with beta-thalassemia shortly after birth and have since been receiving multiple transfusions annually. Two of the HTLV-positive patients confirmed receiving transfusions before HTLV blood screening was implemented in 1988. We identified a substantial HTLV-1 seroprevalence in US thalassemia patients that is much greater than that seen in blood donors. Our findings highlight the importance of HTLV testing of patients with thalassemia and other diseases requiring multiple transfusions, especially in recipients of unscreened transfusions. In addition, appropriate counseling and follow-up of HTLV-infected patients is warranted.

